# Molecular insights into *Aedes aegypti* (L.) populations and vector surveillance in the urban areas of Jeddah and Jizan, Saudi Arabia

**DOI:** 10.3389/finsc.2025.1638582

**Published:** 2025-09-11

**Authors:** Shatha I. Alqurashi, Saad Murya Alqahtani, Khalid M. S. Alghamdi, Somia Eissa Sharawi, Habeeb Mansour Al-Solami, Abdullah G. Alghamdi, Hanan S. Alyahya, Hayat S. Al-Rashidi, Jazem A. Mahyoub

**Affiliations:** ^1^ Department of Biological Science, College of Science, University of Jeddah, Jeddah, Saudi Arabia; ^2^ Department of Biology Sciences, Faculty of Sciences, King Abdulaziz University, Jeddah, Saudi Arabia; ^3^ Department of Biology, College of Science, Jazan University, Jazan, Saudi Arabia; ^4^ Department of Biology, College of Science, Qassim University, Buraydah, Saudi Arabia

**Keywords:** *Aedes aegypti*, Jeddah, Jizan, molecular insights, vector borne disease, dengue fever, phylogenetic

## Abstract

**Introduction:**

The *Aedes aegypti* constitutes the primary vector for dengue fever, yellow fever, chikungunya, Zika, West Nile, and encephalitis viruses, all of which have impacted One Health (human, animal, and environmental health) significantly. It has been distributed widely in urban settings in Saudi Arabia, particularly in cities like Jeddah and Jizan, a situation that underscores the urgent need for innovative and sustainable vector control strategies. Molecular tools, such as DNA barcoding using mitochondrial markers, have become essential for identifying mosquito species accurately and understanding their role in disease transmission. Such knowledge is vital for informing effective, climate-resilient public health interventions.

**Methods:**

This research focuses on identifying *Aedes* species in various regions of Saudi Arabia using polymerase chain reaction (PCR) and sequencing techniques, in order to evaluate the molecular diversity of these dengue vectors in Jeddah and Jizan. The study utilizes the cytochrome one oxidase (COI) gene as a molecular marker for phylogenetic analysis to compare the populations of *Aedes* species.

**Results:**

The findings reveal the presence of significant genetic variation among mosquito populations. In the Jeddah region, the *Ae. aegypti* types MN299016.1 and KU495081.1 match completely (100%) the respective populations found in Argentina and Australia, with 93.1% (27/29) and 6.9% (2/29) respectively. Meanwhile, the samples from the Jizan region are 100% and 99.6% similar to the *Ae. aegypti* types MN298998.1, MK300226.1, PP892777.1, and MF043259.1 found in Canada, Kenya, India, and England.

**Conclusion:**

This study underscores the necessity of using molecular techniques in vector surveillance to mitigate the spread of Zoonotic and vector borne diseases in Saudi Arabia. Moreover, these efforts align with the objectives of the Saudi Vision 2030 by promoting environmentally responsive vector surveillance in Jeddah and Jizan, thereby supporting integrated approaches to public health and ecological sustainability.

## Graphical Abstract

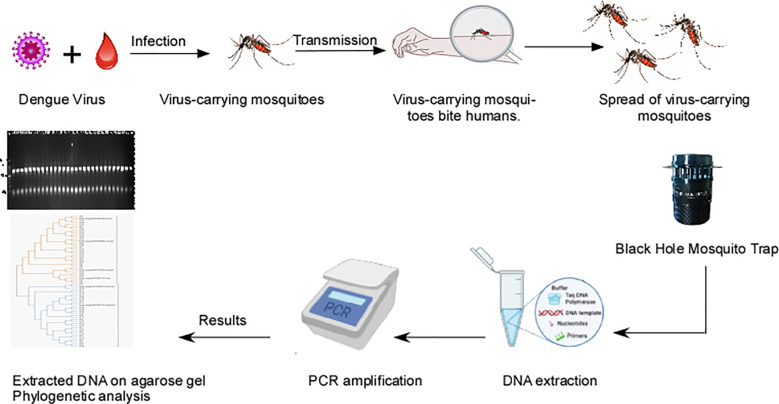

## 1 Introduction

The noxious *Ae. aegypti* inflicts a plethora of vector-borne diseases, with yellow fever, chikungunya, Zika, West Nile, encephalitis viruses, and dengue fever responsible for significant morbidity and mortality in developing countries (1–3). These viral haemorrhagic fevers are prevalent where the urban-dwelling mosquito *Ae. aegypti* is incriminated as a major culprit (4, 5). There are several factors that contribute to the proliferation of mosquitoes and the spread of diseases in Jeddah and Jazan, including temperature, coastal fog, and rainfall (6, 7). In Saudi Arabia, some studies indicate a relationship between climatic conditions and the increase in the number of mosquitoes and their ability to transmit diseases (8), recorded a high density of mosquitoes in Jeddah during the winter at temperatures below 28°C and a lower density in the summer at temperatures of 40°C.

In recent years, some Saudi Arabian cities have seen an epidemic of dengue fever, which are endemic in certain cities of Saudi Arabia, such as Jeddah and Jizan (9). However, there is currently no comprehensive and authentic record of the *Aedes* mosquito species of Jeddah and Jizan, Saudi Arabia.

Detailed reports on mosquito species in Saudi Arabian are available in the literature (10–14). Understanding the diversity and distribution of these species is crucial for research on biodiversity and disease management. It is important to comprehend their habitats and the pathogens they transmit to develop effective vector control strategies aimed at preventing disease transmission (15, 16). Globally, only 10% of mosquito species have been identified, primarily due to a lack of taxonomic expertise (17, 18). The mitochondrial cytochrome c oxidase subunit 1 (COI) gene has demonstrated effective in the rapid and accurate identification of *Aedes aegypti* mosquitoes and their taxonomic features (19). The COI gene is particularly valuable for evolutionary studies (20). Molecular techniques are preferred for identifying species, assessing genetic diversity, and studying molecular evolution because they provide high resolution. These methods do not depend on the sex or age of mosquitoes to produce reliable results (21–23).

According to research conducted recently on insect taxonomy and phylogenetic analysis, DNA barcoding is an effective and convenient method for identifying various genera and species (24–27). In order to identify mosquito species accurately, taxonomically molecular biology using polymerase chain reaction (PCR) was employed by recent researchers (28). Interest in studying mosquitoes from a genetic perspective began in the mid-1950s, especially concerning the species that transmit epidemic diseases, notably those that belong to the genera *Aedes, Anopheles*, and *Culex* (29). The present study investigates the genetic diversity of *Ae. aegypti*, and the vector of dengue fever in the cities of Jazan and Jeddah in Saudi Arabia, using the cytochrome one oxidase (COI) gene as a significant marker. The aim of the study is to examine the COI sequences from random samples of adult *Ae. aegypti* collected from Jazan and Jeddah, in order to determine the similarities/diversity in their DNA sequences, and to compare them with those of the same species from other regions around the world.

## 2 Methods and materials

### 2.1 DNA extraction

All genomic DNA extraction experiments were conducted in molecular identification experimental unit, in the Dengue Mosquito Experimental Station (DMES), belonging to the Department of Biological Sciences, Faculty of Sciences, King Abdul-Aziz University, Jeddah, Saudi Arabia. As (30) explained, DNA can be extracted from preserved mosquito specimens by immersing the entire body of each specimen in absolute ethanol. The insect lysis buffer involved is prepared using the following chemical components: 16.5 grams of GuSCN (Sigma), 12ml of 0.5 M EDTA pH 8.0, 6ml of 1 M Tris-HCL pH 8.0, 1ml of Triton X-100 (Sigma), and 10ml Tween-20 (Fluka). In order to conduct the experiment in the present study, mosquito samples were placed in Eppendorf tubes, and a combination of proteinase K and insect lysis buffer was added to each tube. After vortexing and incubating the samples at 56 °C for 10 minutes, the samples were centrifuged before the supernatant was transferred to a tube for further analysis. The next step was to gently mix the supernatant with 100 litres of ethanol, and then to add the ethanol to the supernatant. Thereafter, the mixture was placed onto a silica gel-based spin column for further separation. In order to remove any possible remaining liquid, we spun the solution at 12,000 RPM for two minutes at the highest speed. The liquid was removed from the collection tube by hand.

In the same experiment, 500 litres of 70% ethanol were used in the process, and the results were the same. Afterwards, the samples were centrifuged again at 12,000 RPM on the spin column for two more minutes before they were removed from the centrifuge for further processing. It was then rewashed with ethanol to remove any remnants of the liquid that remained. To ensure that the spin column had dried completely when the washing was finished, we centrifuged it for three minutes at 13,000 RPM. In order to move the upper part of the spin column to the Eppendorf tube, the upper part of the column was removed. During the release of the DNA that was trapped within the silica gel, it was first necessary to add 20 litres of nuclease water to the centre of the gel and to leave it for about 10 minutes, so that the DNA could be released. A one-minute spin at 12,000 RPM was then applied to the sample, and it was then dried. This was repeated after another 50 litres of nuclease water was added to the solution. The DNA was then eluted from the silica gel and transferred to the Eppendorf tube, after the gel had been turned (30). Using Thermo Fisher Scientific’s NanoDrop 7000 instrument from Waltham, MA, USA, we assessed the quantity and quality of the DNA samples using the A260/Ratio, which was calculated using the NanoDrop 7000. In addition, an electrophoresis of DNA in 1% gel of agarose was conducted to assure the integrity of the DNA. Following the extraction and preservation of the DNA at a temperature of 20 °C for a short period of time, it was then stored at a temperature of -20 °C for a period, after which it could be used for experiments following preservation.

### 2.2 Agarose gel preparation

A microwaveable flask was filled with 70mL 1xTBE buffer, into which was dissolved 0.5% of agarose. The agarose was boiled for 1–2 minutes by swirling the flask occasionally until the agarose was completely dissolved. In order to accomplish this process, ethidium bromide (EtBr) was added to the agarose solution at a final concentration of approximately 0.2-5g/mL after the solvent had warmed up to around 50 °C for about 2–3 minutes. As the gel melted, it was poured into the tray of mini-gel, with the well comb positioned slowly in the middle, in order to avoid causing bubbles in the gel. After 20–30 minutes, once the gel had completely solidified, the comb was removed carefully, and the gel was prepared for use. In addition to covering the gel in the gel box, 1xTBE buffer was also used to load the DNA into each well. One Kbp DNA ladder was also added to each well.

### 2.3 Gene amplification

The mitochondrial cytochrome oxidase subunit I (COI) gene was utilized to identify polymorphism among the different populations used in this study. Gene amplification was preformed using a PCR with the universal barcoding primer set, LCO1490 (5’-GGTCAACAAATCATAAAGATATTGG-3’) and HCO2198 (5’-TAAACTTCAGGGTGACCAAAAAATC-3’) (31), generating amplicons ranging between 600–700 base pairs. Each 25 μl reaction contained 15.3 μl of 1× bovine serum albumin (BSA), 2.5 μl of 10× Reaction Buffer, 2 μl of dNTPs (2.5 μM), 1.25μl of each primer (10 μM/L), 0.2 μl Taq DNA Polymerase (1.0 U), and 2.5 μl of template DNA (>50 ng). The PCR conditions consisted of an initial denaturation at 94 °C for two minutes, followed by 40 cycles of denaturation at 94 °C for 30 seconds, annealing at 49 °C for 45 seconds and extension at 72 °C for 45 seconds, with a final extraction step at 72 °C for one minute. The PCR products were verified by electrophoresis on 1.5% agarose gel and the gel was then analysed under UV light. The amplified bands were subsequently purified from the gel and submitted for sequencing to Macrogen Company, Korea, using an applied Biosystems 3730Xl DNA Analyzer.

### 2.4 Sequencing

For sequencing, the samples were sent to a Macrogen company in Seoul, South Korea. A QIA quick PCR purification kit (Promega, Madison, WI, USA) was used to purify the amplified DNA for sequencing, and a Big Dye terminator cycle sequencing Ready Reaction kit (Applied Biosystems, Forster City, CA, USA) was used to perform the sequencing on an ABI Prism^®^ 310 Genetic Analyzer (Applied Biosystems) to measure the sequence of the amplified DNA.

### 2.5 Sequence and phylogenetic analysis

In order to compare the nucleotide information that was extracted using the sequences discussed above within the Gene Bank database, we used the Basic Local Alignment Search Tool (BLAST) (https://blast.ncbi.nlm.nih.gov/Blast.cgi), which can be found at http://blast.ncbi.nlm.nih.gov/Blast.cgi. A nucleotide sequence alignment was conducted by utilizing the Clustal W tool in MEGA to align the raw nucleotide sequences. In order to construct the phylogenetic tree of each locus, a multiple sequence alignment was conducted using MEGA11 software18, and the sequences were then matched with those in the GenBank database of NCBI (http://www.ncbi.nlm.nih.gov), and the reference sequences were obtained to perform a phylogenetic analysis on the sequences retrieved. A phylogenetic tree of the populations was constructed based on the dissimilarity values computed using the Maximum-Likelihood method with the Tamura-Nei Model2. The distance matrix determined via this approach was utilized to infer the evolutionary relationships across the populations. In addition to the bootstrap analysis, a maximum of 1,000 replicates of the machine learning analysis was also conducted.

## 3 Results and discussion

### 3.1 Molecular identification

**Figure 1:**
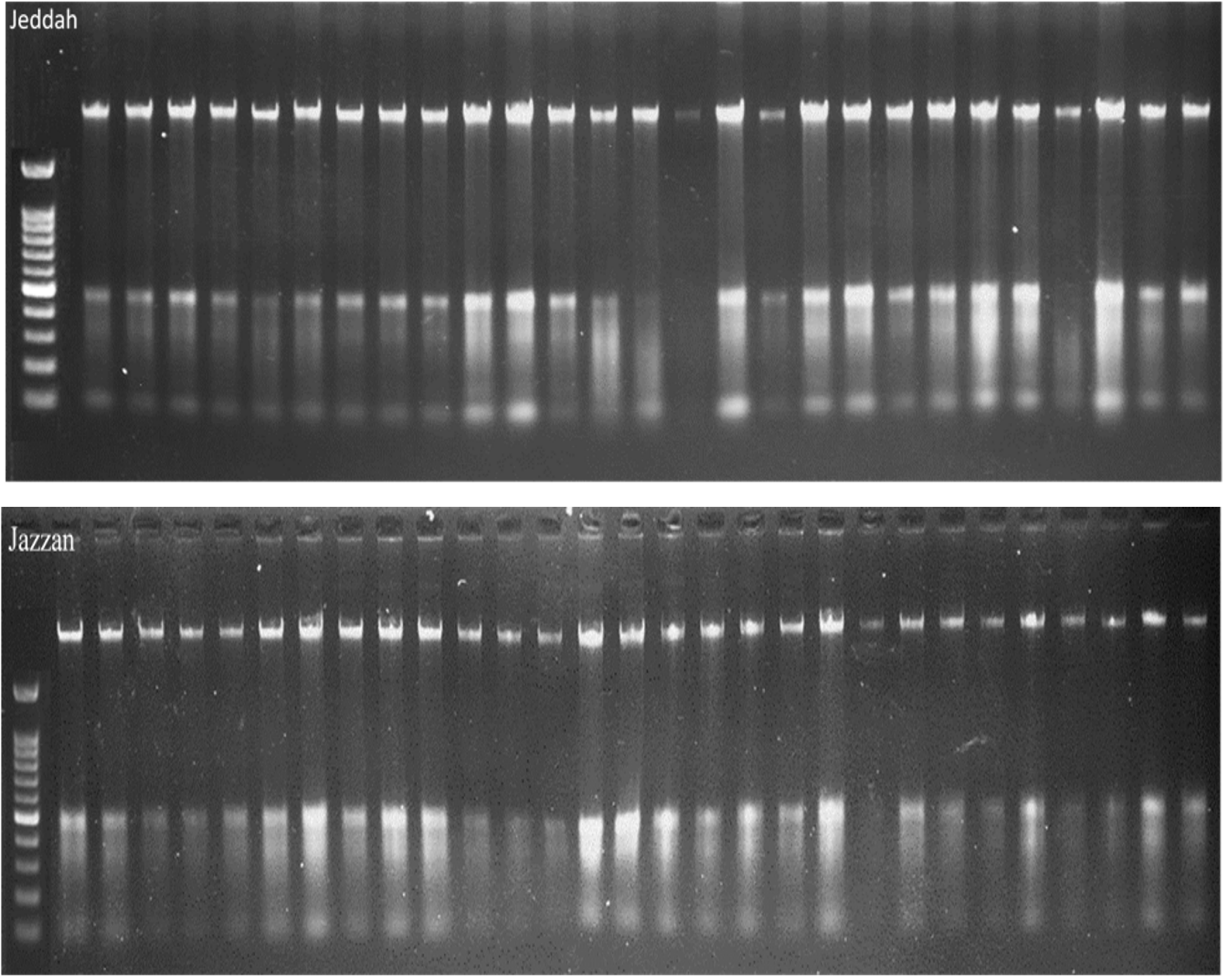
DNA extraction visualized on an agarose gel.

**Figure 2:**
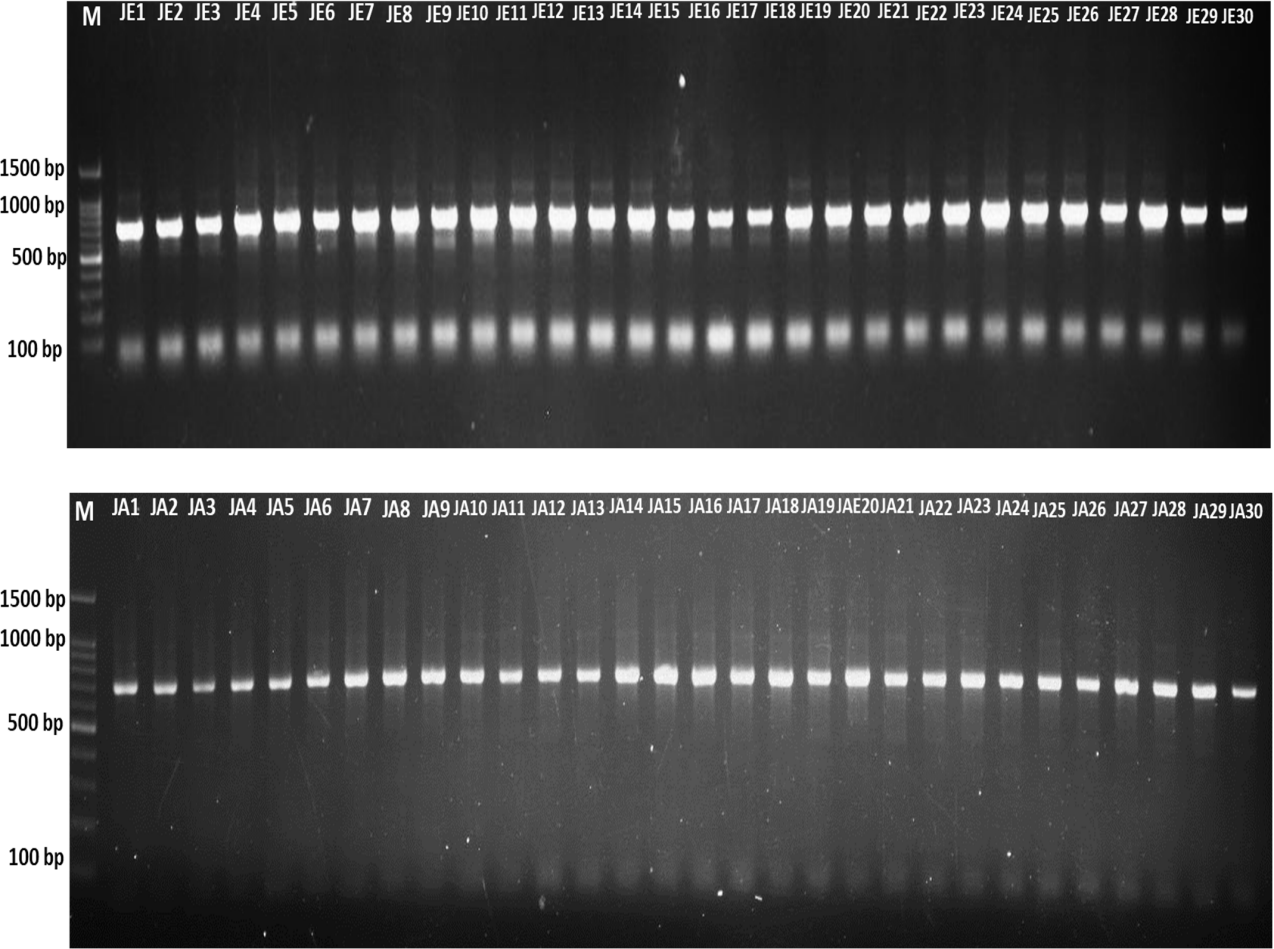
PCR amplification of the mT-COI gene was achieved in 48 mosquito samples. JE, Jeddah; JA, Jazan.

The DNA samples employed for this study were collected from two different regions, Jeddah and Jazan, and consisted of 29 samples from each region, with a total of 58 samples for both regions. By using the same protocol and procedure as that employed by (30), namely lysis buffers, DNA was extracted from the insects and analysed on a 1% agarose gel (**Figure 1**). As a preliminary step towards identification of the gene encoding, Cytochrome c oxidase subunit, I (COX1), genomic DNA was extracted from 58 randomly selected mosquito specimens taken from the two geographical regions in question. As a result of the PCR amplification using universal primers targeting the mTCOX-1 gene, 600–700 bp fragments were generated, which were then subjected to 1-5% agarose gel electrophoresis and then analysed (**Figures 1**, **2**).

### 3.2 Gene sequencing and phylogenetic tree analysis

**Figure 3:**
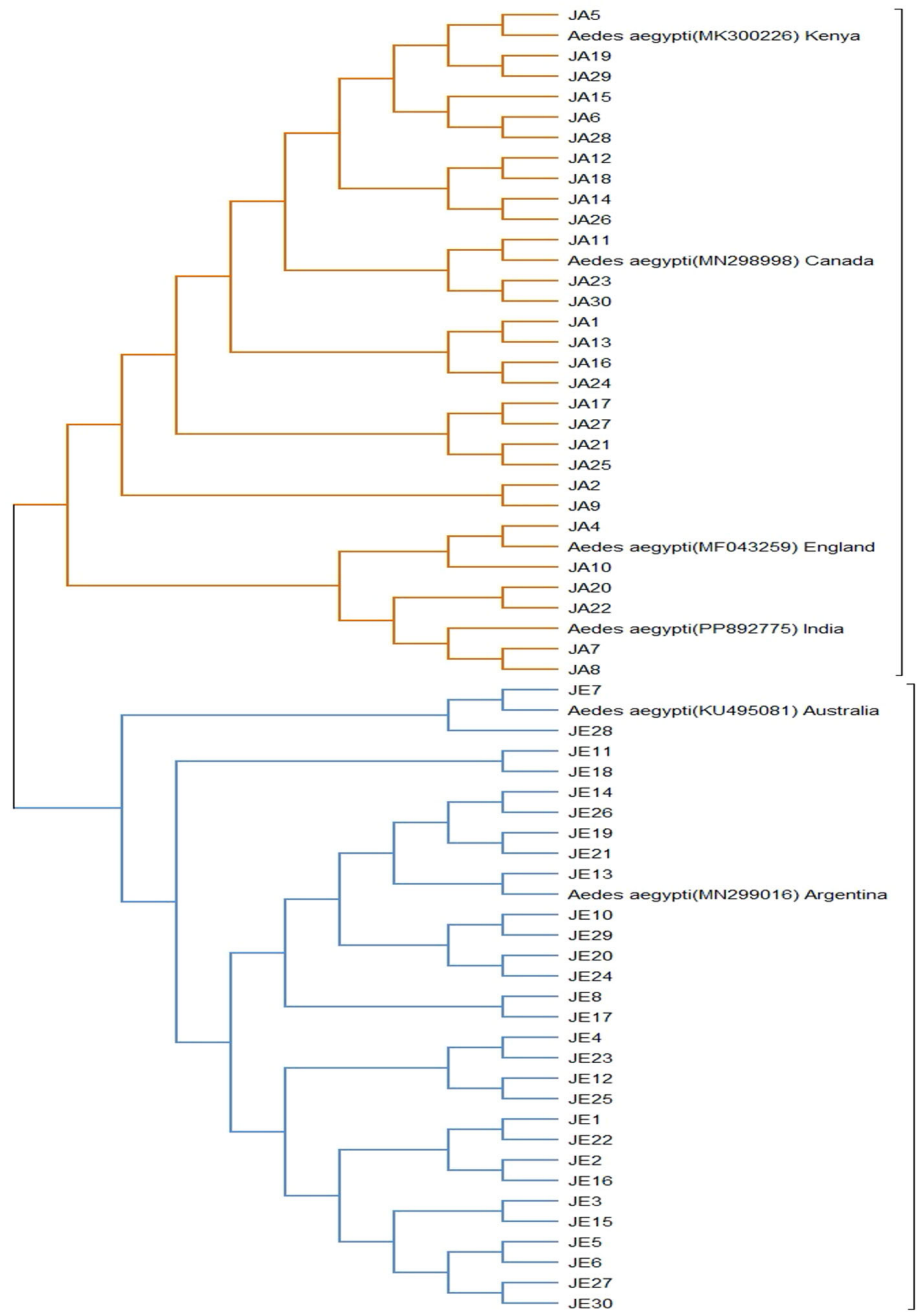
Phylogenetic analysis of the study samples was performed using the Maximum Likelihood (ML) method, as implemented in the MEGA 11 program.

Phylogenetic trees (**Tables 1**, **2**) show the convergence and divergence of the samples (**Figure 2**). Correlations were made between the samples and the gene bank samples (**Table 1**). In the results obtained from the Jeddah region, the *Ae. aegypti* types MN299016.1 and KU495081.1 demonstrated a complete match (100%) with the respective populations found in Argentina and Australia, with 93.1% (27/29) and 6.9% (2/29), respectively (**Table 1**; **Figure 3**). Meanwhile, in the samples from the Jazan region, the samples were 100% and 99.6% like the *Ae. aegypti* types MN298998.1, MK300226.1, PP892777.1, and MF043259.1 found in Canada, Kenya, India, and England (**Table 1**; **Figure 3**), with proportions of 58.6% (17/29), 20.7% (6/29), 13.8% (4/29), and 6.9% (2/29), respectively. As shown in **Figure 3**, the evolutionary distances between the taxa examined were compared with each other, and with the sequences related to the NCBI, in order to determine where the samples evolved from each other. A phylogenetic tree created with MEGA11 represented the convergence and divergence among the samples analysed. The phylogenetic analysis of the mTCOX-1 gene revealed that the species from Jeddah and Jazan did not match each other; instead, they formed two different clusters, according to the results of the analysis, which showed that the *Ae. aegypti* species formed two major groups.

**Table 1:** Comparing study samples with NCBI Gene Bank datasets.

No. samples	Genebank code	Gene published date	Organism and gene definition	Gene length (bp)	Pre % Idnt	Country	References
Jeddah							
**JE1,JE2, JE3, JE4, JE5, JE6, JE8, JE10, JE11, JE12, JE13, JE14, JE15, JE16, JE17, JE18, JE19, JE20, JE21, JE22, JE23, JE24, JE25, JE26, JE27, JE29, JE30**	**MN299016.1**	09-JUN-2020	*Ae. aegypti isolate PeCO2 cytochrome oxidase subunit I (COI) gene, partial cds; mitochondrial*	701 bp	100%	Argentina	Lapadula, W. J., Marcet, P. L., Taracena, M. L., Lenhart, A., & Ayub, M. J. (2020). Characterization of horizontally acquired ribotoxin encoding genes and their transcripts in Aedes aegypti. Gene, 754, 144857 (32).
**JE7, JE28**	**KU495081.1**	25-MAY-2016	*Ae. aegypti voucher VAITC4689 cytochrome oxidase subunit 1 (COI) gene, partial cds; mitochondrial*	687 bp	100%	Australia	Batovska, J., Blacket, M. J., Brown, K., & Lynch, S. E. (2016). Molecular identification of mosquitoes (Diptera: *Culicidae*) in southeastern Australia. Ecology and Evolution, 6(9), 3001-3011 (33).
Jazan							
**JA1,JA2, JA9,JA11, JA12, JA13, JA14, JA16, JA17, JA18, JA21, JA23, JA24, JA25, JA26, JA27, JA30**	**MN298998.1**	07-AUG-2019	*Ae. aegypti isolate RDC7 cytochrome oxidase subunit I (COI) gene, partial cds; mitochondrial.*	668 bp	100	Canada	Lapadula, W. J., Marcet, P. L., Taracena, M. L., Lenhart, A., & Ayub, M. J. (2020). Characterization of horizontally acquired ribotoxin encoding genes and their transcripts in *Ae. aegypti*. Gene, 754, 144857 (32).
**JA5, JA6, JA15, JA19, JA28, JA29**	**MK300226.1**	14-DEC-2018	*Ae. aegypti isolate NAH11 cytochrome c oxidase subunit 1 (COI) gene, partial cds; mitochondrial*	709 bp	100%	Kenya	Makanda, M., Wahome, L., Onyambu, G., Mutunga, J. and Bargul, J. (2018). Direct Submission. Institute of Basic Sciences Technology and Innovation, The Pan African University, Nairobi, Kenya (34)
**JA7,JA8, JA20, JA22**	**PP892777.1**	14-JUN-2024	*Ae. aegypti isolate AA2 cytochrome c oxidase subunit I (COX1) gene, partial cds; mitochondrial*	646 bp	99.69%	India	Nayak,B., Sahu,B., Pattanayak,A.K. and Barik,T.K. (2024). Mitochondrial COX1 sequence of *Ae. aegypti.*PG Department of Zoology, Berhampur. University, Bhanjabihar, Berhampur, Odisha 760007, India (39)
**JA4, JA10**	**MF043259.1**	12-JUN-2017	*Ae. aegypti voucher BMNH: ENT : BM9 cytochrome oxidase subunit I (COI) gene, partial cds; mitochondrial*	695 bp	100%	England	Dallimore, T., Hunter, T., Medlock, J. M., Vaux, A. G., Harbach, R. E., & Strode, C. (2017). Discovery of a single male *Ae. aegypti* (L.) in Merseyside, England. Parasites & Vectors, 10, 1-8 (40).

Bold values indicate GenBank accession numbers, unique identifiers in the NCBI database that link to complete nucleotide records.

**Table 2:** The molecular identification of new collected *Ae. aegypti* from the Jeddah and Jazan regions and their accession numbers.

No.	Isolate code	Scientific name	Our accession number	Accession number of NCBI	Similarity (%)
1	JE1	*Ae. aegypti*	PQ098152	MN299016.1	100%
2	JE2	*Ae. aegypti*	PQ098153	MN299016.1	100%
3	JE3	*Ae. aegypti*	PQ098154	MN299016.1	100%
4	JE4	*Ae. aegypti*	PQ098155	MN299016.1	100%
5	JE5	*Ae. aegypti*	PQ098156	MN299016.1	100%
6	JE6	*Ae. aegypti*	PQ098157	MN299016.1	100%
7	JE7	*Ae. aegypti*	PQ098158	KU495081.1	100%
8	JE8	*Ae. aegypti*	PQ098159	MN299016.1	100%
9	JE10	*Ae. aegypti*	PQ098161	MN299016.1	100%
10	JE11	*Ae. aegypti*	PQ098162	MN299016.1	100%
11	JE12	*Ae. aegypti*	PQ098163	MN299016.1	100%
12	JE13	*Ae. aegypti*	PQ098164	MN299016.1	100%
13	JE14	*Ae. aegypti*	PQ098165	MN299016.1	100%
14	JE15	*Ae. aegypti*	PQ098166	MN299016.1	100%
15	JE16	*Ae. aegypti*	PQ098167	MN299016.1	100%
16	JE17	*Ae. aegypti*	PQ098168	MN299016.1	100%
17	JE18	*Ae. aegypti*	PQ098169	MN299016.1	100%
18	JE19	*Ae. aegypti*	PQ098924	MN299016.1	100%
19	JE20	*Ae. aegypti*	PQ098170	MN299016.1	100%
20	JE21	*Ae. aegypti*	PQ098171	MN299016.1	100%
21	JE22	*Ae. aegypti*	PQ098172	MN299016.1	100%
22	JE23	*Ae. aegypti*	PQ098173	MN299016.1	100%
23	JE24	*Ae. aegypti*	PQ098174	MN299016.1	100%
24	JE25	*Ae. aegypti*	PQ098175	MN299016.1	100%
25	JE26	*Ae. aegypti*	PQ098176	MN299016.1	100%
26	JE27	*Ae. aegypti*	PQ098177	MN299016.1	100%
27	JE28	*Ae. aegypti*	PQ098178	KU495081.1	100%
28	JE29	*Ae. aegypti*	PQ098179	MN299016.1	100%
29	JE30	*Ae. aegypti*	PQ098180	MN299016.1	100%
30	JA1	*Ae. aegypti*	PQ098181	MN298998.1	100%
31	JA2	*Ae. aegypti*	PQ098182	MN298998.1	100%
32	JA4	*Ae. aegypti*	PQ098183	MN299016.1	99.78%
33	JA5	*Ae. aegypti*	PQ098184	MK300226.1	100%
34	JA6	*Ae. aegypti*	PQ098185	MK300226.1	100%
35	JA7	*Ae. aegypti*	PQ098186	PP892777.1	99.69%
36	JA8	*Ae. aegypti*	PQ098187	PP892777.1	99.69%
37	JA9	*Ae. aegypti*	PQ098188	MN298998.1	100%
38	JA10	*Ae. aegypti*	PQ098189	MN299016.1	100%
39	JA11	*Ae. aegypti*	PQ098190	MN298998.1	100%
40	JA12	*Ae. aegypti*	PQ098191	MN298998.1	100%
41	JA13	*Ae. aegypti*	PQ098192	MN298998.1	100%
42	JA14	*Ae. aegypti*	PQ098193	MN298998.1	100%
43	JA15	*Ae. aegypti*	PQ098194	MK300226.1	100%
44	JA16	*Ae. aegypti*	PQ098195	MN298998.1	100%
45	JA17	*Ae. aegypti*	PQ098196	MN298998.1	100%
46	JA18	*Ae. aegypti*	PQ098197	MN298998.1	100%
47	JA19	*Ae. aegypti*	PQ098198	MK300226.1	100%
48	JA20	*Ae. aegypti*	PQ098199	PP892777.1	99.69%
49	JA21	*Ae. aegypti*	PQ098200	MN298998.1	100%
50	JA22	*Ae. aegypti*	PQ098201	PP892777.1	99.69%
51	JA23	*Ae. aegypti*	PQ098202	MN298998.1	100%
52	JA24	*Ae. aegypti*	PQ098203	MN298998.1	100%
53	JA25	*Ae. aegypti*	PQ098204	MN298998.1	100%
54	JA26	*Ae. aegypti*	PQ098205	MN298998.1	100%
55	JA27	*Ae. aegypti*	PQ098206	MN298998.1	100%
56	JA28	*Ae. aegypti*	PQ098207	MK300226.1	100%
57	JA29	*Ae. aegypti*	PQ098208	MK300226.1	100%
58	JA30	*Ae. aegypti*	PQ098209	MN298998.1	100%

This study found that samples from Jazan demonstrated genetic similarity with those from Canada, Kenya, England, and India, which constituted the first cluster of samples. As a result of a further subdivision within this cluster, it was observed that there were two distinct sections, the first of which contained only the samples from Canada and Kenya, and the second contained two elements. In the first clade, the samples that matched those found in England were exclusively located, while the second clade contained samples that matched those found in India. As a result of the differentiation between the two clades, subclades were identified. The samples collected from the city of Jeddah formed part of the second cluster, as they demonstrated a genetic similarity with those from Argentina and Australia. In addition to the subdivision within this cluster, two distinct sections were identified within the cluster. Those sampled in the first section could only be compared with those from Argentina, whereas those from the second section were comparable with those from Australia. Several genetic mutations were observed in the study samples, which could be considered the cause of the differences observed between them.

This study aimed to molecularly identify *Ae. aegypti* mosquitoes from two distinct regions in Saudi Arabia: Jeddah and Jazan. By utilizing the mt COX1 gene as a marker, phylogenetic and sequence analyses revealed significant genetic variability between the mosquito populations from these regions. The results contributed valuable insights into the genetic structure of *Ae. aegypti*, providing data that may influence vector control programmes, given the importance of genetic diversity in disease transmission and control strategies.

### 3.3 Molecular identification of *Ae. aegypti*

The molecular identification of mosquito samples using the COX1 gene marker conducted by this study proved effective for distinguishing *Ae. aegypti* populations in Jeddah and Jazan. In both regions, all of the samples were identified as *Ae. aegypti*, confirming the species’ presence in these areas, as expected, due to its widespread distribution in tropical and subtropical regions (32, 33). The genetic variation observed between the two regions underscored the species’ adaptability to different environmental conditions.

The DNA extraction and PCR amplification conducted yielded fragments of 600–700 bp, corresponding to the COX1 gene, which was sequenced successfully. The sequences obtained were compared with those available in the NCBI Gene Bank, confirming the samples’ species as *Ae. aegypti* (**Tables 1**, **2**). These findings corroborated previous reports regarding the utility of the COX1 gene as a molecular marker for species-level identification in mosquitoes (30).

### 3.4 Phylogenetic analysis and genetic divergence

A phylogenetic analysis using the Maximum Likelihood (ML) method was conducted, which revealed that there were two distinct genetic clusters; these corresponded to the mosquito populations from Jeddah and Jazan (**Figure 3**). This genetic divergence could be attributed to geographic isolation or to differences in selective pressures between the two regions, such as environmental conditions and human activity. The samples from Jeddah showed a complete match (100%) with *Ae. aegypti* strains from Argentina and Australia (MN299016.1 and KU495081.1, respectively). These samples formed a separate cluster in the phylogenetic tree, suggesting that the Jeddah population shared a closer genetic relationship with the *Ae. aegypti* populations from these regions. This may indicate a common origin or recent introduction, potentially due to human-mediated movement, such as international trade or travel (32). In contrast, the samples from Jazan displayed a more complex pattern of genetic similarity, matching *Ae. aegypti* strains from Canada, Kenya, India, and England (MN298998.1, MK300226.1, PP892777.1, and MF043259.1, respectively). This population formed two distinct sub-clusters, suggesting greater genetic diversity within the Jazan population. This may be the result of genetic introgression from multiple sources, or historical population expansion and contraction in response to environmental changes (34).

### 3.5 Implications of genetic divergence

The genetic divergence observed between the Jeddah and Jazan populations has important implications for mosquito control programmes. Genetic variation within mosquito populations can affect their susceptibility to insecticides and their ability to transmit diseases, such as dengue, Zika, and chikungunya. For example, populations with distinct genetic backgrounds may respond differently to chemical interventions, requiring tailored approaches for effective control (33). The divergence between the Jazan and Jeddah clusters suggested that control strategies may be required to be region-specific.

Although the present study did not analyse specific insecticide resistance genes, previous studies have documented resistance associated kdr mutations in *Ae. aegypti* populations from Jeddah and other regions of Saudi Arabia (35–37). These mutations, especially those in the voltage-gated sodium channel (Vssc) gene, have been strongly linked to pyrethroid resistance. A broader regional context displays the emergence of “super-resistance” due to combined kdr mutations in parts of Asia, posing serious challenges for vector control (38). These findings underscore the importance of incorporating insecticide resistance markers into future genetic analyses. This will help align population dynamic with resistance patterns, enhancing the accuracy and impact of region-specific vector control efforts.

For instance, the Jazan population’s genetic similarity to *Ae. aegypti* from geographically distant regions, such as Canada and Kenya may reflect a broader genetic pool that could influence vector competence or insecticide resistance. This highlighted the need for ongoing surveillance and molecular monitoring to track the genetic changes in mosquito populations and the need to adjust control strategies accordingly (39).

### 3.6 Geographic and environmental influence

The differences in genetic clustering between the Jeddah and Jazan populations may also reflect the geographic and environmental conditions of these regions. Jeddah, which is a coastal city, experiences higher humidity and temperature levels than Jazan, which may influence selective pressures on mosquito populations. Environmental factors, such as temperature, humidity, and vegetation, have been shown to affect mosquito breeding, survival, and behaviour, which in turn can shape genetic diversity (40). Furthermore, the human population density and urbanization in Jeddah may play a role in the genetic similarity of its mosquito population to those in Argentina and Australia, as urbanization tends to homogenize genetic diversity, due to the increased movement of people and goods. In contrast, the more rural setting of Jazan, with its agricultural activities, may provide a more heterogeneous environment, contributing to the greater genetic diversity observed in the mosquito population in this region.

## 4 Conclusion

This study successfully utilized molecular identification and phylogenetic analysis to investigate the genetic variation of the *Aedes aegypti* populations collected from Jeddah and Jazan, Saudi Arabia. The results revealed substantial genetic divergence between the two populations, emphasizing the complex population structure of this important disease vector within the different geographic regions of the Kingdom. Notably, the Jeddah population demonstrated a closer genetic relationship with populations from Argentina and Australia, suggesting a possible introduction pathway or a shared ancestral lineage. In contrast, the Jazan population exhibited a higher degree of genetic diversity with genetic similarities to mosquitoes from Canada, Kenya, India, and England, indicating a potentially more diverse origin or greater gene flow in this region. These findings highlight the need for region-specific mosquito control strategies. Genetic variability, both within and between populations, can significantly influence vector competence, insecticide resistance, and the overall effectiveness of control measures. Therefore, understanding the genetic structure of local mosquito populations is essential for designing targeted and sustainable vector control programs. This study highlights the importance of continuous genetic surveillance by using molecular tools. Monitoring the genetic dynamics of *Ae. aegypti* populations over time will be vital for tracking evolutionary trends, identifying potential resistance developments, and adapting public health interventions accordingly. In the context of Saudi Arabia, integrating molecular data into vector control policies will enhance the precision and success of mosquito management efforts, especially in high-risk urban areas, such as Jeddah and Jazan.

## Data Availability

The datasets presented in this study can be found in online repositories. The names of the repository/repositories and accession number(s) can be found in the article/Supplementary Material.
